# Building a learning culture and prevention of error – to near miss or not

**DOI:** 10.1002/jmrs.242

**Published:** 2017-09-06

**Authors:** Anthony Arnold

**Affiliations:** ^1^ Illawarra Shoalhaven Local Health District Wollongong Australia

## Abstract

This editorial provides an insight into learning and prevention of error through near miss event reporting.

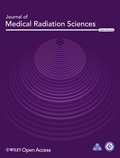

The preparation and delivery of radiation therapy involves multiple and often complex procedures that are performed by three professional groups, radiation therapists, radiation oncologists and medical physicists. While this intricate complexity exists, there is a high expectation of safety in radiation therapy. Identification of medical error and near miss events through incident reporting systems and the subsequent correction and preventative actions can improve clinical practice, process and workflow and patient safety, this is nothing new.^1^ Reviews of previous incidents and events have identified that having a system for reporting incidents may be a possible way to prevent an incident from reoccurring.^2^

It is widely reported and understood, that in a radiation oncology, the majority of errors are minor in nature, whereas the rare major incidents are typically not an isolated event, there will always be many factors involved, and almost always it will have developed over time through some systematic failure affecting many patients at a time. Herein, lies the importance of not only reporting incidents – but building a culture that supports openness and focus on learning and prevention.

In the broad healthcare setting, incident reporting and management is not performed well. The system naturally reverts to a retrospective analysis with too often a focus on the individuals and not the systematic faults behind the event/s in question. This culture is discouraging for staff and the motivation for ongoing reporting is lost.

Based on my extensive experience designing and implementing radiation oncology incident reporting, the staff proactively report, learn and prevent future incidents. Most if not all radiation oncology centres in Australia and New Zealand have a system for reporting, collating and analysing errors and near misses. It could be that the very process driven workflow that are performed by the radiation oncologists, radiation therapists and medical physicists contributes to the willingness to report.

Radiation therapy technology is continually evolving at a rapid pace hence the established quality assurance (QA) checkpoints must be redesigned to ensure the continuation in the detection of error. The reporting of near misses provides a wealth of information that can be used to support data driven change in the workflow.

In the development of a radiation oncology specific reporting system whilst working at Liverpool Cancer Therapy Centre between 2004 and 2007 we analysed the reporting data across the first 3 years of using the system.^3^ Of all the reports over that time (688), over 75% of them (533) were near miss reports. Of those near miss reports, 65% (348) were detected at fixed quality assurance checkpoints throughout the system – designed specifically for this purpose.

Now some will argue that those near miss reports should not be included in an analysis as they detected at the fixed checkpoints designed for that purpose. I would and will advocate the opposite. Those 348 near miss reports provide invaluable insight into the workflow and quality of process. Each and every one of them is an opportunity to improve the system, so that the checkpoint no longer detects that event.

Every quality assurance system is striving to get to a point where zero error is detected and the check is somewhat redundant. Further, having the 348 reports included provides insight into the pressures that each particular check point was under. We should all be using this data to improve the quality and structure of upstream processes so that over time discrepancies are ideally eliminated or reduced.

This type of reporting and learning from the near miss data drives the quality improvement cycle, referred to as PDSA (Plan, Do, Study, Act). Staff reports the event, and if the culture is right, own the event as together they contribute to recommendations and preventative ideas which are trialled and reviewed. The incident or report itself is a moment in time. The learning and preventative actions that evolve over time are what turns those moments into momentum. The momentum that is created will continue to drive improvements in patient safety.

In 2005, the Tripartite Committee, which is a peak group in radiation oncology representing the three key professions involved in radiation therapy; The Royal Australian and New Zealand College of Radiologists (RANZCR), Faculty of Radiation Oncology (FRO), Australian Society of Medical Imaging and Radiation Therapy (ASMIRT) and the Australasian College of Physical Scientists and Engineers in Medicine (ACPSEM) received Australian Government funding support for the development and publication in 2011 of the Radiation Oncology Practice Standards.^4^ Standard 14 relates to the incident monitoring program, and provides supplementary data for facilities to classify events in a consistent manner, irrespective of the jurisdictional or local system they may be using. This standard provides the ability for a facility to classify their data in terms of event class, dosimetric error level, and clinical consequence specific to the radiation oncology workflow.

A key element in successful analysis of the reported data is of course the incident reporting taxonomy itself. Typical healthcare based reporting systems are not refined to a level of detail to classify radiation oncology workflow. If reports are analysed against a consistent taxonomy such as that identified in the practice standards, then the potential to pool data and develop greater insight exists on a much larger scale.

Denham and Page 5 in their paper in this journal issue have demonstrated the power in a much broader analysis of reported events. In radiation oncology specifically, the potential exists to develop a national repository of nationwide data, extracted from the numerous systems across the country. It is this ultimate goal that many like myself in the profession continue to work towards. The true system wide learning and prevention of error through near miss reporting would be taken to the next level.

Radiation therapy is becoming more and more reliant on automation. These automated processes and automated data still need to be validated and checked appropriately. Many facilities are working on automated upstream quality assurance checks and systems at key points in the process. While undoubtedly these will help, we cannot forget that there are still humans at the end of the line, contouring tumours, creating margins and targets for treatment, interpreting pre‐treatment verification images and delivering lifesaving radiation treatment itself.

Building the right culture, with an openness to report and be transparent about error and near miss will improve the learning culture within a facility. Harnessing that learning culture and building a focus on prevention will reduce errors. Reducing errors and lowering the error rate over time, leads directly to improved patient safety, which is always and should be at the centre of what we do.
